# Probiotic *Bacillus coagulans* GBI-30, 6086 Improves Protein Absorption and Utilization

**DOI:** 10.1007/s12602-017-9354-y

**Published:** 2017-12-01

**Authors:** Ralf Jäger, Martin Purpura, Sean Farmer, Howard A. Cash, David Keller

**Affiliations:** 1 Increnovo LLC, 2138 E Lafayette Pl, Milwaukee, WI 53202 USA; 2 Ganeden Probiotics, 5800 Landerbrook Dr, Suite 300, Mayfield Heights, OH 44124 USA

**Keywords:** *Bacillus coagulans*, Probiotic, Protein, Nutrient absorption, Intestinal microbiota

## Abstract

Probiotics offer numerous health benefits, including digestive and immune health. Improved digestive health is linked to a more efficient absorption of important nutrients from our diet. This review focused on the rationale of using the probiotic *Bacillus coagulans* GBI-30, 6086 to aid protein absorption and utilization. *B. coagulans* GBI-30, 6086 can withstand the acidic environment of the stomach to reach the intestine where it germinates. Once active in the small intestine after germination, it has been shown to aid the digestion of carbohydrates and proteins. Co-administration of *B. coagulans* GBI-30, 6086 with protein has been shown to increase protein absorption and to maximize the health benefits associated with protein supplementation.

## Introduction

Probiotics are live microorganisms that, when administered in adequate amounts, confer a health benefit on the host [1]. Probiotics modulate the growth or survival of bacteria in the gut lumen, improve mucosal barrier function, and affect the mucosal and systemic immune system [2]. The large intestine is the most heavily colonized organ in the human body, and stress, diet, infections, living conditions, and antimicrobial intake can negatively affect the microflora composition [3]. Probiotics administration beneficially influences nutrient absorption and availability. Probiotics can optimize mineral absorption possibly through changes in the pH levels, have major impact on carbohydrate digestion through the production of digestive enzymes, can reduce cholesterol levels through degrading cholesterol in the gut, and can even produce vital nutrients, including the synthesis of various vitamins (for a recent review, see [4]). Efficacy of all probiotics is strain specific, and effects of one strain cannot be assumed on another strain unless proven through clinical trials.

The probiotic *Bacillus coagulans* GBI-30, 6086 (GanedenBC30) [5] is a lactic acid producing, spore-forming bacterial species. Due to the formation of spores, *B. coagulans* can withstand the acidic environment of the stomach to reach the intestine where it germinates. Once active in the small intestine after germination, it has been shown to aid the digestion of carbohydrates and proteins [6]. Consumption of *B. coagulans* GBI-30, 6086 creates an intestinal environment that is not hospitable to various pathogens, creating a healthier and more efficient intestinal tract that is better able to utilize nutrients that have been consumed [7]. In addition to better utilization of consumed foods, *B. coagulans* GBI-30, 6086 increases the benefits of prebiotics by promoting populations of beneficial bacteria as well as the production of short-chain fatty acids essential for the health of cells lining the gut [8]. *B. coagulans* GBI-30, 6086 has been shown to improve dysbiosis by increasing the groups of beneficial bacteria [9]. The modulation of the gut microbiota results in a decrease of abdominal pain and bloating in subjects with irritable bowel syndrome (IBS) [], reduces the number of daily bowel movements in subjects with diarrhea-predominant IBS [11] as well as a decrease gas and bloating and improve quality of life in subjects with post prandial intestinal gas symptoms [12]. The strain has also shown anti-inflammatory and immune modulating effects [13, 14]. *B. coagulans* GBI-30, 6086 has a history of safe use and is safe for chronic human consumption based upon toxicology testing, even when the product is consumed in high quantities [15, 16].

Here, we review the effect of *B. coagulans* GBI-30, 6086 on protein digestion and utilization.

## Increasing Protein and Carbohydrate Metabolism in an In Vitro Gut Model

The survival and the ability to aid the digestion of protein and carbohydrates of *B. coagulans* GBI-30, 6086 was tested in an in vitro model of the stomach and small intestine (TIM-1) [6]. Under the tested condition, the survival of strain was high (70%). *B. coagulans* GBI-30, 6086 produces enzymes that have been shown to aid the breakdown of protein and a wide variety of carbohydrates [5]. The addition of *B. coagulans* GBI-30, 6086 to milk protein increased the amount of digested milk protein available for absorption.

## Increasing Protein Absorption in a Mini-swine Animal Model

*B. coagulans* GBI-30, 6086 has been shown to increase protein utilization in a mini-swine animal model by 10% after 4–5-month-old male mini-swine were fed either a certified diet only or certified diet plus 1 billion CFU *B. coagulans* GBI-30, 6086 per pig per day for 4 weeks.

## Increasing Amino Acid Appearance in the Blood in Humans

*B. coagulans* produce digestive enzymes that are active under gut conditions (alkaline proteases, etc.) and these proteases have been shown to increase amino acid appearance in the blood when co-administered with protein [17]. In addition, *B. coagulans* GBI-30, 6086 enhances the health of the cells of the gut lining by decreasing inflammation, thereby improving nutrient absorption through optimum development of the absorptive area of the villi [14]. The benefits of *B. coagulans* GBI-30, 6086 on protein absorption overserved in the in vitro model and an animal model were confirmed in a randomized, double-blind, placebo-controlled, crossover human clinical trial. Healthy subjects consumed either whey protein or whey protein plus 1 billion CFU *B. coagulans* GBI-30, 6086 for 2 weeks. *B. coagulans* GBI-30, 6086 increased absorption of amino acids of specific importance to athletes and people interested in muscle health, including BCAAs (leucine, isoleucine, valine), or amino acids involved in blood flow regulation, such as citrulline, or recovery (glutamine).

## Increasing Muscle Health in Athletes: Reduced Exercise-Induced Muscle Damage and Increased Recovery

Skeletal muscle mass is the sum of muscle protein breakdown (MPS) and muscle protein synthesis (MPS). The protein kinase mechanistic target of rapamycin (mTOR) has been widely recognized as a key regulator of MPS and muscle mass. Mechanical stimulation of mTOR through weight-bearing exercise, or dietary activation through protein ingestion both stimulate MPS and are additive when protein consumption follows exercise. The recommended daily protein intake to maintain nitrogen balance (muscle mass) is 0.8 g protein/kg body weight/day, however, people trying to increase muscle mass should increase their daily protein intake to 1.4–2.0 g protein/kg body weight/day. Optimal protein intake per serving for people trying to maximize MPS are 20–25 g of a high-quality protein, containing 2–3 g of leucine, to be consumed every 3–4 h. Ingestion of higher amounts of protein does not further stimulate MPS. Rapidly digested proteins that contain high proportions of essential amino acids (EAA), of which the key amino acid appears to be leucine, are more effective in stimulating MPS than other proteins [18].

**Fig. 1:**
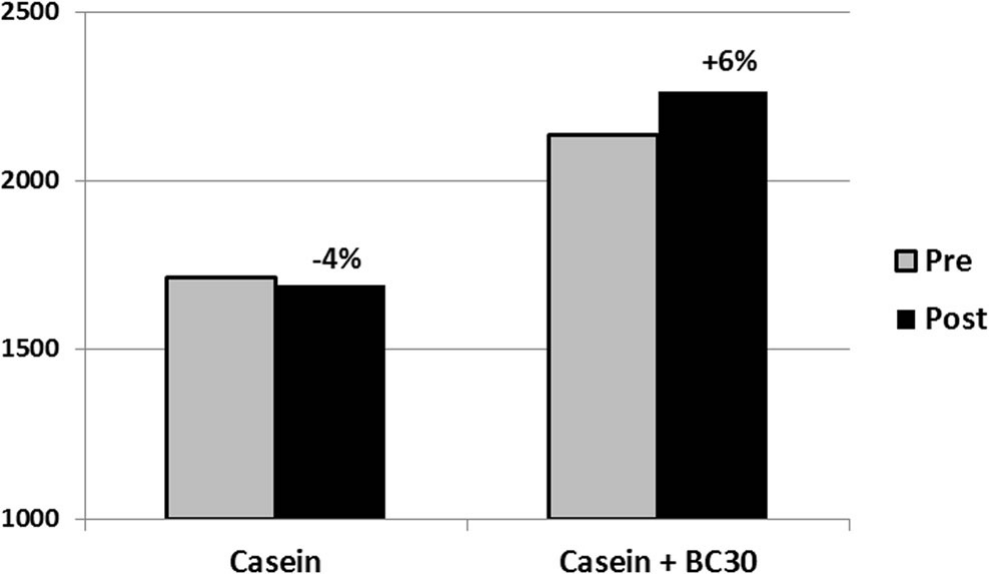
Co-administration of 20 g casein and 500 million CFU BC30 (*B. coagulans* GBI-30, 6086) twice daily tended to increase vertical jump peak power over casein alone following an 8-week resistance training program [19]

Post-workout administration of slow digesting proteins such as casein show suboptimal results on MPS in comparison to fast absorbed proteins such as whey. *B. coagulans* GBI-30, 6086 has been shown to increase EAA absorption, including leucine absorption by 20%. The potential beneficial effects of co-administration of the probiotic *B. coagulans* GBI-30, 6086 with a slow digested protein on body composition and sports performance has been studied in a pilot trial [19]. Healthy resistance-trained individuals consumed either 20 g of casein or 20 g of casein plus 500 million CFU *B. coagulans* GBI-30, 6086 twice daily in combination with a resistance training program, consisting of full body workouts 4 times per week for 8 weeks. The addition of *B. coagulans* GBI-30, 6086 showed a trend (*p* = 0.10) to increase vertical jump power in comparison to casein alone, and might had a beneficial effect on peak power and fat mass (Fig. 1).

There were no significant differences between groups for body composition, or other performance measures. This pilot study indicated that probiotic supplementation in form of *B. coagulans* GBI-30, 6086 in combination with a slow digesting protein trended to increase athletic performance.

**Fig. 2:**
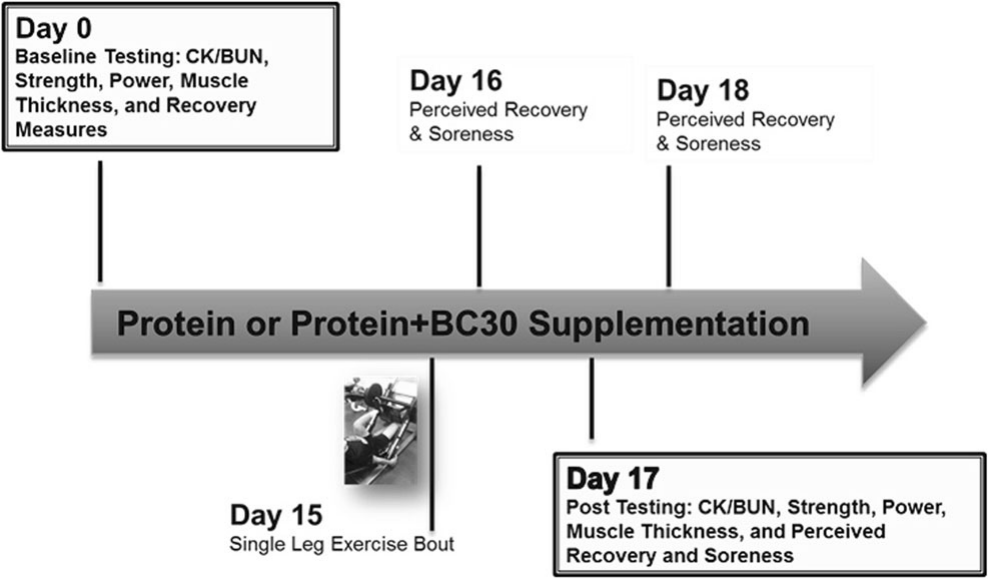
Study design investigating the effects of *B. coagulans* GBI-30, 6086 following a muscle-damaging exercise [20]

The beneficial effects observed in vertical jump performance might be based on aiding muscle recovery through gut microbial modulation. A follow-up study investigated if the co-administration of *B. coagulans* GBI-30, 6086 with protein has beneficial effects on muscle damage, performance, and recovery following a muscle-damaging exercise bout [20]. Recreationally trained males consumed 20 g casein, and following a 1 week wash-out period 20 g of casein plus 1 billion CFU *B. coagulans* GBI-30, 6086 daily for 2 weeks. Participants performed a muscle-damaging one-legged exercise bout and perceived recovery, muscle soreness, strength and power, as well as markers of muscle damage and hypertrophy were measured post exercise (Fig. 2).

**Fig. 3:**
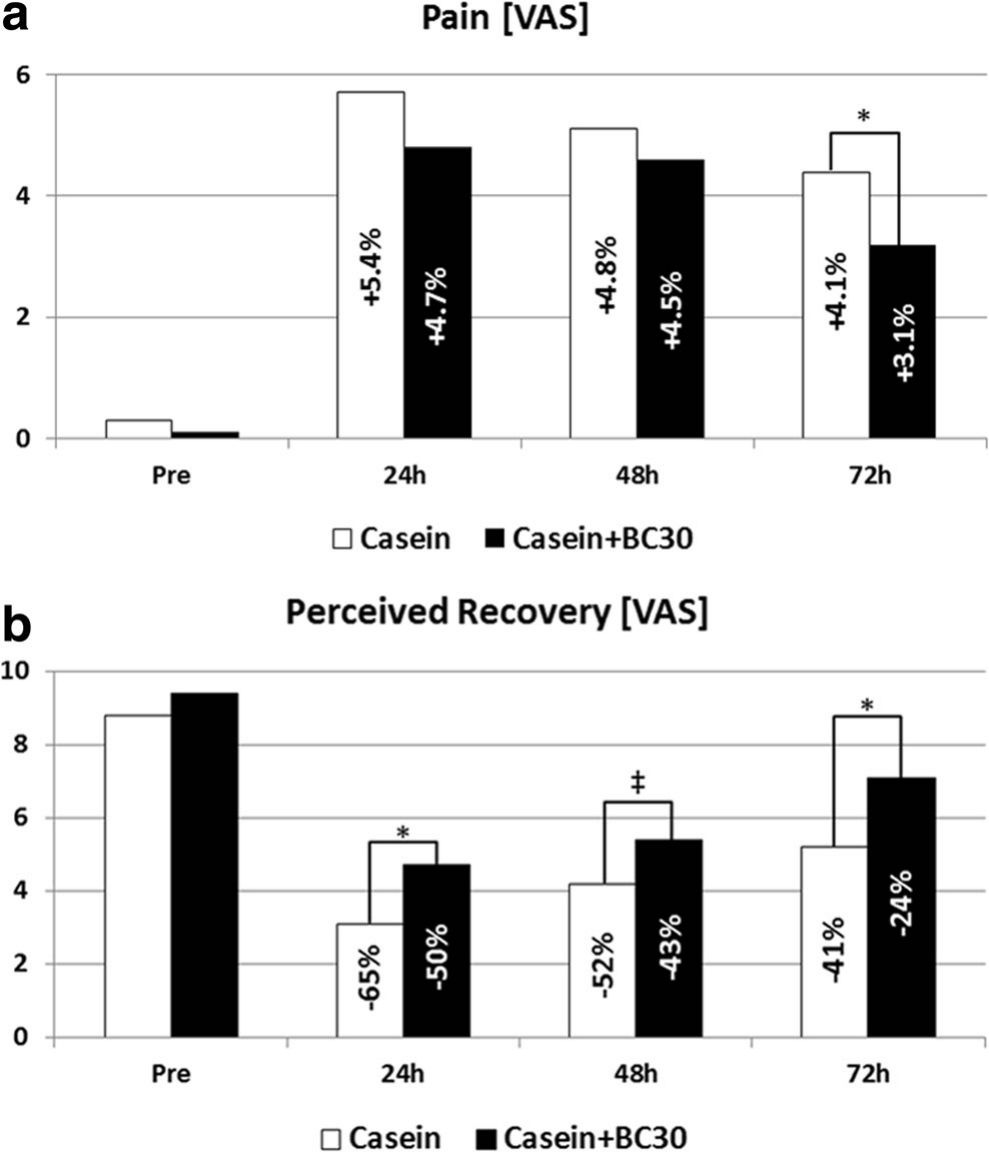
*B. coagulans* GBI-30, 6086 significantly reduced subjected feelings of perceived muscle soreness (left) and increased perceived recovery (right) from muscle-damaging exercise using a visual analogue scale (VAS) [20]

The muscle-damaging exercise significantly increased muscle soreness and reduced perceived recovery in the protein alone group. However, the co-administration of *B. coagulans* GBI-30, 6086 with casein significantly reduced soreness and increased perceived recovery (Fig. 3).

**Fig. 4:**
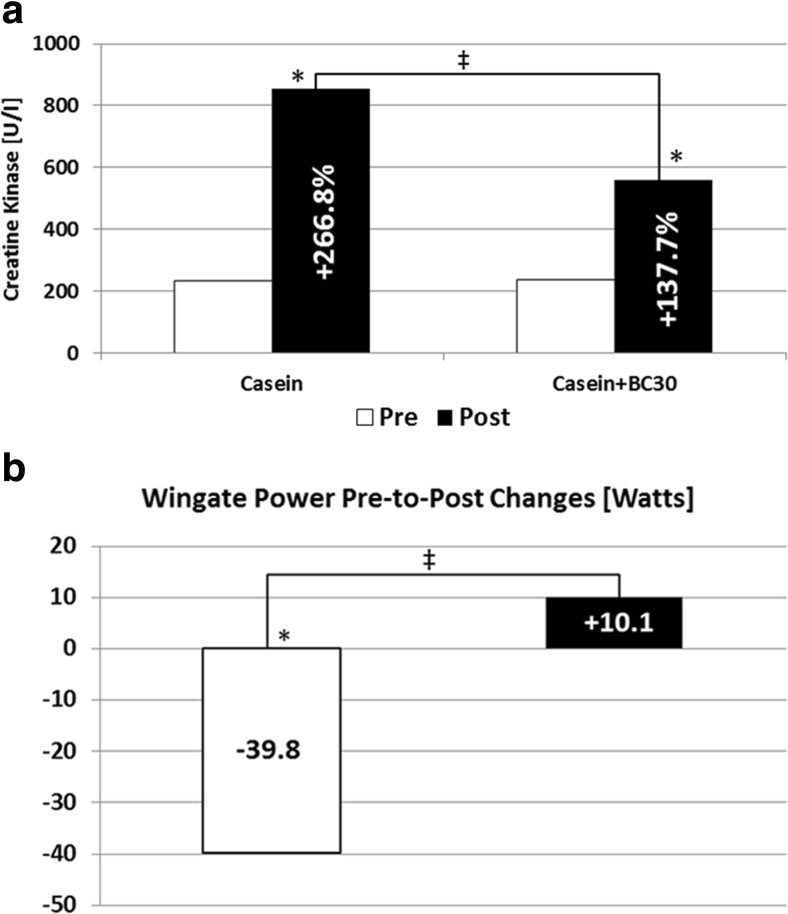
*B. coagulans* GBI-30, 6086 reduces muscle damage (left) and beneficially effects subsequent performance (right) following a bout of muscle-damaging exercise [20]

The subjective measures were confirmed by blood markers of muscle damage. Co-administration of *B. coagulans* GBI-30, 6086 reduced the observed increase in muscle damage. Exercise performed, as measured through the Wingate cycle ergometer test determining peak power, significantly decreased athletic performance in the casein group whereas co-administration of *B. coagulans* GBI-30, 6086 and casein prevented the decline. The addition of *B. coagulans* GBI-30, 6086 to casein did not further improve body composition or strength (Fig. 4).

Reduced muscle damage and faster recovery may lead to an enhanced adaptation rate to training and subsequent to a faster increase in hypertrophy and performance. Exercise-induced skeletal muscle tissue damage occurs as a result of the forced lengthening of active muscle, which directly causes microtears of the myofibrils, resulting in muscle soreness and swelling, and decreased forced production. The initial reaction is followed by a secondary inflammatory response integral to skeletal muscle repair and recovery response. While muscle damage appears to be an important component of muscular adaptation to exercise, a reduction and not a complete prevention of muscle damage, as shown by the addition of *B. coagulans* GBI-30, 6086 to casein, might be optimal.

## Probiotic Health Benefits in Active People

In addition to improved nutrient absorption and production, regular probiotic supplementation has been linked to improved digestive and immune health, both crucial to athletes, as severe exercise has been linked to increased gut permeability and suppressed immune health. Moderate intensity exercise reduces the infection risk, while high-intensity exercise actually increases infection risk. Immune suppression in athletes worsens by psychological stress, environmental extremes, exposure to large crowds, or increased exposure to pathogens due to elevated breathing during exercise [21]. Probiotic supplementation in athletes has been reported to reduce the number, severity, and duration of upper respiratory infections and gastrointestinal distress in athletes [22].

## Concluding Remarks

The probiotic *B. coagulans* GBI-30, 6086 improves protein absorption and utilization and thereby optimizes the health benefits associated with protein supplementation. Based on the improved digestion of protein, *B. coagulans* GBI-30, 6086 could be used to improve muscle health in various populations and reduce quality differences of protein sources naturally lower in leucine, such as plant proteins.
